# Sleeping Sickness

**Published:** 1907-08-24

**Authors:** 


### LIVERPOOL SCHOOL OF TROPICAL MEDICINE.

### SLEEPING SICKNESS.

A considerable amount of work has recently been accomplished in regard to the treatment of sleeping sickness, including under that term the early cases (trypanosomiasis) of the disease. An International Conference recently met in England to discuss the general question of the spread of the disease in Africa and other points, but nothing very definite resulted from the deliberations that took place. It is difficult exactly to see in what way a Conference could control the scientific work that has been and is being done in many of the phases of this disease, and the German delegates expressed themselves on this point somewhat strongly. The credit of the discovery of a drug that has proved more efficacious than any others tried before must go to the Liverpool School of Tropical Medicine. The names associated with the introduction of atoxyl in the treatment of typanosomiasis are those of Thomas and Breinl, while Tod, another worker in the same school, has in frequent articles published from time to time, drawn special attention to the modes of administering it and some of the results obtained. The latter observer in a memorandum by himself, and also later in a combined paper with Breinl, advocates the following method for giving the drug. "Atoxyl must not be given by the mouth, since it is certainly broken up by the acid contents of the stomach, and the untoward effects of over-treatment by arsenic are thus more easily produced. We believe that the intravenous injection of a solution of atoxyl in distilled water is the method to be preferred. Its difficulties are apparent, and in routine treatment it will probably be usually replaced by subcutaneous injections. As in the ordinary administration of arsenic, the organism should be gradually accustomed to the drug. We believe that this can best be done by using a 20 per cent. solution of atoxyl in sterile normal saline. The solution should be warmed to blood heat just before use. In this way the drug is completely dissolved, and the pain at the site of injection, which occasionally follows atoxyl, is obviated. Give subcutaneously daily for four days 0.6 c.cm. On each of the four succeeding days give 0.8 c.cm., then raise the dose for a week to 1 c.cm. of the solution each day. Now give 1 c.cm. every two days for a fortnight. Then reduce to 1 c.cm. twice a week until all symptoms have disappeared and the patient's blood is negative to subcutaneous inoculation into susceptible animals. Afterwards 1 c.cm. should be given weekly for as long a period as possible. Should signs of poisoning arise, the same doses should be given but less frequently." They also point out that it is useless attempting the atoxyl treatment unless the patient is prepared to continue it carefully and intelligently for long periods, and of course at the same time attempts must be made to build up the patient's health by good food, fresh air, and other means. Daniels, quoted by the same authors, states that in two cases of human trypanosomiasis, treated with 10 minim doses of a 10 per cent. solution, the dose gradually being increased to 25 to 30 minims on alternate days, for fourteen and ten months respectively, have now none of the symptoms of the disease, and parasites can no longer be found in their blood. In addition to being in perfect health their blood is no longer toxic to guinea-pigs or rats. Such results are very promising, but, as Breinl and Tod point out, we must beware in concluding that the amelioration, which follows the giving of the drug, is to prove permanent. Several years must elapse before one can say, and the cases must not be lost sight of. Koch is reported to have stated that the drug is as good a specific for sleeping sickness as quinine is for malaria, but, on the other hand, Kopke, though getting good results at first, found that the cases relapsed and died later. Recently Nierenstein, another worker at the Liverpool School of Tropical Medicine, has found that a combination of atoxyl and mercury has given better results on animals than atoxyl alone, and strongly recommends the trial of this combination on human cases. It will be exceedingly interesting to note what results will follow such a treatment, and also to see if someone will come forward with some new drug still better than those which have gone before.

